# High-Yield Soluble Expression and Simple Purification of the Antimicrobial Peptide OG2 Using the Intein System in *Escherichia coli*

**DOI:** 10.1155/2013/754319

**Published:** 2013-07-02

**Authors:** Yong-Gang Xie, Fei-Fei Han, Chao Luan, Hai-Wen Zhang, Jie Feng, Young-Jun Choi, Denis Groleau, Yi-Zhen Wang

**Affiliations:** ^1^Key Laboratory of Animal Nutrition and Feed Science of Ministry of Agriculture, Key Laboratory of Feed and Animal Nutrition of Zhejiang Province, Institute of Feed Science, Zhejiang University, Hangzhou, Zhejiang 310058, China; ^2^Biotechnology Research Institute, National Research Council, Montreal, QC, Canada H4P 2R2; ^3^Chemical and Biotechnological Engineering, University of Sherbrooke, Sherbrooke, QC, Canada J1K 2R1

## Abstract

OG2 is a modified antimicrobial peptide, that is, derived from the frog peptide Palustrin-OG1. It has high antimicrobial activity and low cytotoxicity, and it is therefore promising as a therapeutic agent. Both prokaryotic (*Escherichia coli*) and eukaryotic (*Pichia pastoris*) production host systems were used to produce OG2 in our previous study; however, it was difficult to achieve high expression yields and efficient purification. In this study, we achieved high-yield OG2 expression using the intein fusion system. The optimized OG2 gene was cloned into the pTWIN1 vector to generate pTWIN-OG2-intein2 (C-terminal fusion vector) and pTWIN-intein1-OG2 (N-terminal fusion vector). Nearly 70% of the expressed OG2-intein2 was soluble after the IPTG concentration and induction temperature were decreased, whereas only 42% of the expressed of intein1-OG2 was soluble. Up to 75 mg of OG2-intein2 was obtained from a 1 l culture, and 85% of the protein was cleaved by 100 mM DTT. Intein1-OG2 was less amenable to cleavage due to the inhibition of cleavage by the N-terminal amino acid of OG2. The purified OG2 exhibited strong antimicrobial activity against *E. coli* K88. The intein system is the best currently available system for the cost-effective production of OG2.

## 1. Introduction

In general, antimicrobial peptides (AMPs) are small peptides (10–50 amino acids) with a net positive charge (generally +2 to +9) and a substantial proportion (≥30%) of hydrophobic residues [1]. They are distributed in a wide range of organisms from single-celled microorganisms to humans [2] and play important roles in host immune defense by direct inhibiting of bacteria, fungi, viruses, and parasites growth and by immune modulation. By doing so, AMPs are regarded as a new generation of antibiotics as well as innate immune modulators [1].

Amphibian skin, such as skin of the *Odorrana grahami* where 107 novel AMPs were discovered [3], is one of the most generous sources of AMPs. The mature Palustrin-OG1 (OG1) is one of those peptides that showed high activity against *Escherichia coli* ATCC25922 and *Staphylococcus aureus* ATCC25923 at the concentration of 16 *μ*g/mL [3, 4]. However, the concentration of OG1 that induces 50% hemolysis of human erythrocytes (HC_50_) was 49.6 *μ*g/mL [4], which limits the application of OG1 as a therapeutic agent. In such case, OG2 (KKFFLKVLTKIRCKVAGGCRT) was generated through amino acid deletions and substitutions from the sequence of OG1, and this newly designed OG2 showed higher net positive charge, higher amphiphilicity, and lower hydrophobicity than OG1. Since OG2 showed much lower cytotoxicity and higher antimicrobial activity than the parental peptide OG1, it could be applicable as a therapeutic agent [5].

Heterogonous expression of recombinant peptides in microbes is one of the cost-efficient methods to provide sufficient quantities to investigate structure-function relationships for further development. We tested many strategies to produce OG2 in both *E. coli* and *Pichia pastoris*. Thioredoxin (TrxA) and small ubiquitin-related modifier (SUMO) were used as fusion partners to express OG2 as a fusion peptide in *E. coli*. However, SUMO-OG2 formed inclusion bodies, whereas TrxA-OG2 was poorly cleaved by enterokinase, and the peptide released by tobacco-etch virus protease degraded quickly [6]. Furthermore, the expression rate of His-tagged OG2 in *P. pastoris* was extremely low (unpublished). Compared with the traditional enzymatic removal of fusion tags, self-cleaving systems such as the intein system are attractive since they simplify the purification process to a single chromatographic step with the adjustment of the reaction temperature and pH value or the addition of small-molecule redox agent such as dithiothreitol (DTT) [7]. 

In this study, we used the intein fusion system to express OG2 as a soluble form in the prokaryotic host and purified OG2 in a single step process. The two inteins encoded by genes in the pTWIN1 vector, *Synechocystis* sp. DnaB (intein1) and *Mycobacterium xenopi *GyrA (intein2), were fused with OG2 to generate intein1-OG2 (N-terminal fusion) and OG2-intein2 (C-terminal fusion), respectively. Both constructs were expressed, purified, and compared with one another.

## 2. Materials and Methods

### 2.1. Construction of Expression Vectors

**Figure 1:**
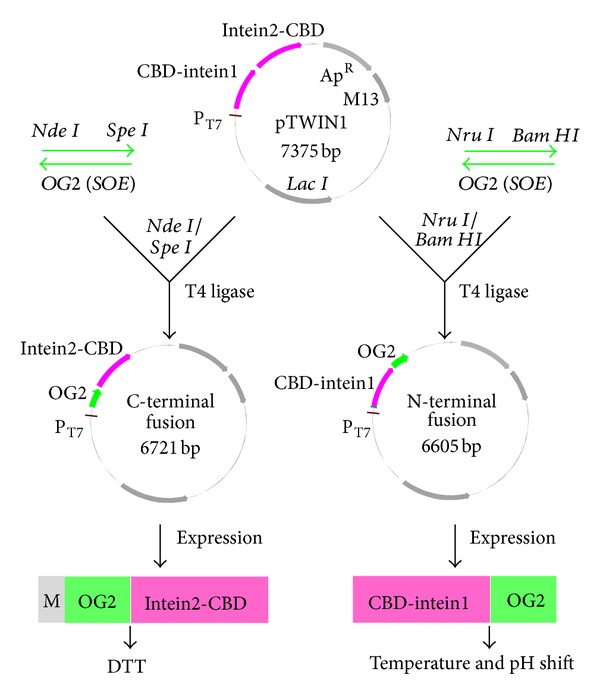
Schematic representation of the vector constructions and the expression of the N-terminal fusion and C-terminal fusion proteins. In the C-terminal fusion protein, intein2-CBD was fused to the C-terminus of OG2, allowing the cleavage of OG2 from OG2-intein2 using DTT. In the N-terminal fusion protein, CBD-intein1 was fused to the N-terminus of OG2, allowing the cleavage of OG2 from intein1-OG2 through a pH and temperature shift.

The two codon-optimized *OG2* genes including *C-OG2* (C-terminal fusion expression) gene and *N-OG2* (N-terminal fusion expression) gene were amplified using splitting overlap extension (SOE) PCR and cloned into the pTWIN1 (NEB, USA) vector (Figure 1). The primers and restriction enzymes used are shown in Table 1. Restriction sites (underlined) were introduced in the first and the last primers. The stop codon TAA was introduced in PN3.

Amplification was carried out in 50 *μ*L volume containing 2 *μ*M PC1 (PN1) and PC3 (PN3) and 0.1 *μ*M PC2 (PN2). The reaction condition was as follows: 97°C for 5 min, 30 cycles of 95°C for 30 s, 60°C for 30 s and 72°C for 1 min, and a final extension of 72°C for 10 min. PCR products were double-digested with the corresponding enzymes and cloned into a pTWIN1 vector that had been digested with the same enzymes. The positive recombinant plasmids were confirmed by sequencing.

### 2.2. Optimization of Protein Expression

Each of recombinant plasmids were transformed into *E. coli* BL21(DE3) pLysS (Novagen, USA), and the resulting positive colonies were designated BL21-OG2-intein2 (C-terminal fusion expression) and BL21-intein1-OG2 (N-terminal fusion expression), respectively. For each recombinant strain, a single colony was inoculated in 3 mL LB medium supplemented with antibiotic agent ampicillin (100 *μ*g/mL) and incubated at 37°C. Approximately 0.5 mL overnight culture was inoculated into 50 mL LB medium, and protein expression took place under different induction conditions (Table 2). For OG2-intein2, cells were harvested and suspended in buffer C1 (20 mM Tris pH 8.5, 0.5 M NaCl, 0.2% (v/v) Tween 20 and 10% (v/v) glycerol). For OG2-intein2, cells were suspended in buffer N1 (20 mM phosphate pH 8, 0.5 M NaCl, 0.2% (v/v) Tween 20, and 10% (v/v) glycerol). Cells were then lysed by two passes through a French press at 1000 psi. After centrifugation at 30,000 ×g for 20 min at 4°C, both the supernatant and the cell debris (inclusion body) fractions were subjected to electrophoresis on a NuPAGE Novex 4–12% Bis-Tris gel (Invitrogen, USA). The gel was stained with Coomassie G-250 SimplyBlue SafeStain (Invitrogen, USA) and distained with double distilled water. The gel image was analyzed by Quantity One software (Bio-Rad, USA). The protein concentration of the supernatant was determined using the Bradford method using bovine serum albumin as the standard protein.

### 2.3. Small-Scale Purification and On-Column Cleavage

Both strains were induced under optimized conditions based on the data obtained from our preliminary research. Cells from 50 mL of culture were harvested and suspended in 5 mL buffer C1 or N1. The cells were lysed and centrifuged as described above. Purification was conducted with the AKTA purifier system. For soluble protein purification, the supernatant was loaded onto a 1 mL chitin (NEB, USA) column equilibrated with C1 or N1. Nonspecifically bound proteins were removed by washing with 20 mL of C1 or N1 and a subsequent wash with 30 mL of high-salt buffer C2 (20 mM Tris pH 8.5, 2 M NaCl, and 0.2% (v/v) Tween 20) or N2 (20 mM phosphate pH 8, 2 M NaCl, and 0.2% (v/v) Tween 20). The cleavage of OG2-intein2 was induced by DTT while that of intein1-OG2 was induced by pH and temperature shift. Therefore, the column was flushed quickly with 5 mL cleavage buffer C3 (20 mM Tris pH 9, 0.5 M NaCl, 40 mM, or 100 mM DTT) or N3 (20 mM phosphate pH 6, and 0.5 M NaCl), and subsequently incubated at 25°C for 24 h. The released OG2 was eluted using cleavage buffer without DTT, and the fusion fragment and uncleaved fusion protein that remained bound to the resin were eluted with 2% SDS. Both the purified peptide and the eluted fusion fragment were analyzed by gel electrophoresis, as described above.

For intein1-OG2, we also attempted to recover and purify the inclusion body fraction. Cells were induced with 0.5 mM IPTG at 37°C overnight. The inclusion bodies were dissolved in 20 mM Tris pH 8 plus 8 M urea overnight, concentrated with a Microcon YM-3 (3 kDa) tube, and dialyzed against refolding buffer (20 mM Tris pH 8, 0.1 mM oxidized glutathione, 1 mM reduced glutathione, 0.5 M L-Arg, 0.2% (v/v) Tween 20, and 5% (v/v) glycerol). The solubilized inclusion bodies were purified using the same method described above.

### 2.4. Large-Scale Expression and Purification

Cells in 1 L culture were induced under the optimized condition, suspended in 100 mL C1 or N1 buffer and lysed using a French press. The fusion protein was purified by 10 mL chitin from approximately 20 mL supernatant each time. After on-column cleavage and elution, both the 2% SDS elution and the peptide elution fractions were subjected to electrophoresis on a NuPAGE Novex 4–12% Bis-Tris gel. The released peptide was desalted with a Sephadex G10 column using 5 mM NH_4_HCO_3_. The eluate was then lyophilized and dissolved in double-distilled water. The peptide concentration was determined with the Bradford method using the chemically synthesized OG2 as the standard peptide.

### 2.5. Antimicrobial Assay

Both agar diffusion test and modified broth microdilution method were used to evaluate the antimicrobial activity of expressed OG2. For the Oxford cup agar diffusion, the sample was added to an Oxford cup, which was then placed on a Mueller-Hinton agar plate containing 10^5^ colony-forming units of *E. coli* K88. Chemically synthesized OG2 was used as a positive control. The minimal inhibition concentration was determined by modified broth microdilution method which was described before [8].

## 3. Results

### 3.1. Gene Cloning and Construction of Expression Vectors

Bands of 111 bp and 102 bp, the expected sizes of the* C-OG2* and *N-OG2* genes, respectively, were obtained by SOE PCR (data not shown). DNA sequencing confirmed the correct insertion of the target genes.

### 3.2. Optimization of Protein Expression

**Figure 2:**
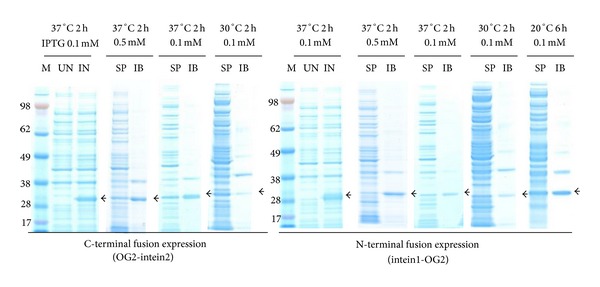
Optimization of protein expression. lane M: SeeBlue Plus2 Pre-Stained Standard (kDa); lane UN: uninduced culture; lane IN: induced culture; lane SP: soluble protein; lane IB: inclusion body.

Protein bands of approximately 30.3 kDa and 27.5 kDa, corresponding to the molecular weights of OG2-intein2 and intein1-OG2, respectively, were observed on the SDS-PAGE gel after induction (Figure 2, shown by arrows). Only 33% of the expressed OG2-intein2 was soluble after induction with 0.5 mM IPTG at 37°C. When the IPTG concentration was decreased to 0.1 mM and the induction temperature was lowered to 30°C, nearly 70% of the expressed OG2-intein2 became soluble. Although a low temperature (20°C) and a low IPTG concentration (0.1 mM) also enhanced the solubility of intein1-OG2, approximately 58% of the protein remained as insoluble. Therefore, induction with 0.1 mM IPTG for 2 h at 30°C and induction with 0.1 mM IPTG for 6 h at 20°C were used in the following experiments as optimum conditions for expression of OG2-intein2 and intein1-OG2, respectively.

### 3.3. Small-Scale Purification and On-Column Cleavage

**Figure 3:**
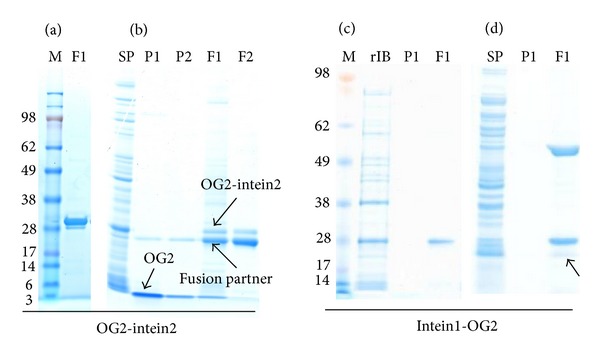
Purification, and on-column cleavage of the fusion proteins. OG2-intein2 was purified using a 1 mL chitin column and cleaved by incubation with 40 mM DTT for 24 h (a) or 100 mM DTT for 24 h (b). Both the recovered insoluble (c) and soluble intein1-OG2 (d) were purified using a 1 mL chitin column, and cleavage was induced by shifting the pH from 8 to 6 and changing the temperature from 4°C to 25°C. lane M, SeeBlue Plus2 Pre-Stained Standard (kDa); lane SP, soluble protein; lane P, released OG2 (P1 and P2 were two fractions of 0.6 mL each); lane F, proteins eluted from the chitin column with 2% SDS (F1 and F2 were two fractions of 0.6 mL each); lane rIB, recovered inclusion body.

For OG2-intein2, only a small proportion of the fusion tag was released under the condition of 40 mM DTT treatment (Figure 3(a)). The cleavage efficiency was greatly improved when the DTT concentration was increased to 100 mM (Figure 3(b)), and most of the fusion tag was released after a 24 h reaction (Figure 3(b), lanes F1 and F2), producing a clear band corresponding to the molecular weight of the OG2 (Figure 3(b), lanes P1 and P2). The other band in the peptide elution lane is the fusion partner, which may be present due to the overloading of the fusion protein sample.

For intein1-OG2, both the inclusion body and soluble protein were subjected to purification. The inclusion bodies were recovered (Figure 3(c), lane rIB) and purified (Figure 3(c), lane F1) by chitin affinity chromatography, but the purified protein underwent little cleavage after a 24 h reaction at pH 6 and 25°C (Figure 3(c)). The soluble fusion protein in the supernatant was isolated successfully (Figure 3(d), lane F1); however, a protein of approximately 60 kDa, possibly the *E. coli* host chaperone protein GroEL, was copurified. Furthermore, this protein was poorly cleaved (Figure 3(d), lane F1, indicated by an arrow).

### 3.4. Large-Scale Expression and Purification

**Figure 4:**
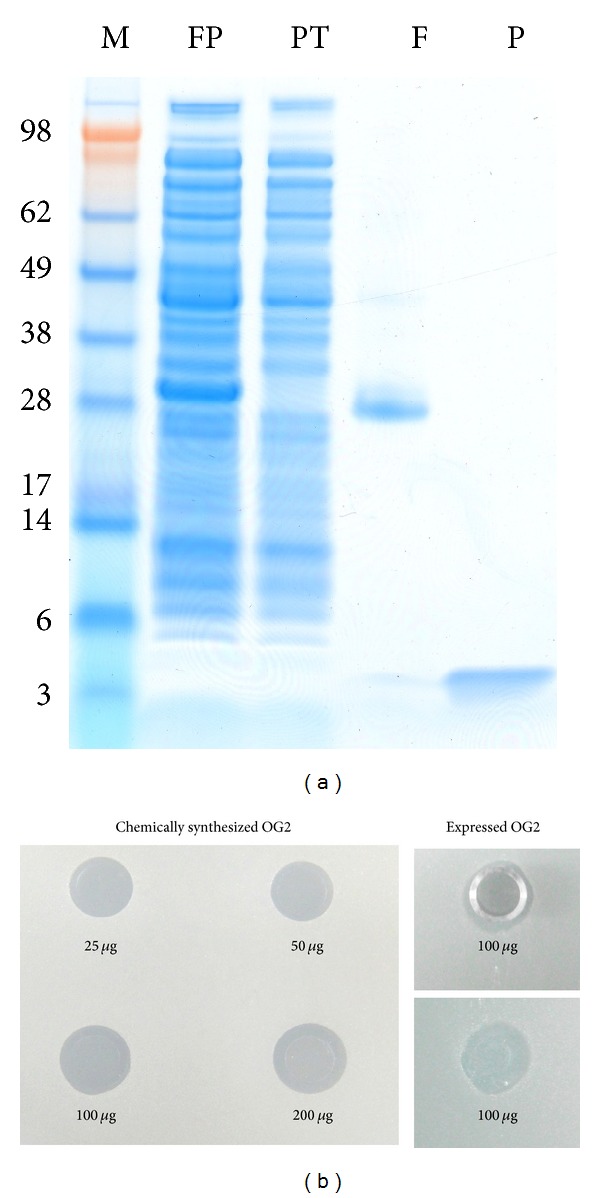
Large-scale expression, purification, and activity of OG2. (a) Purification (10 mL chitin) and on-column cleavage of OG2-intein2. lane M, SeeBlue Plus2 Pre-Stained Standard (kDa); lane SP, soluble protein; lane FT: flowthrough; lane F, proteins eluted from the chitin column with 2% SDS; lane P, released OG2. (b) Comparison of the antimicrobial activities of chemically synthesized OG2 (left) and expressed OG2 (right). For expressed OG2, both the halo with Oxford cup (top) and the halo without the cup (bottom) are shown.

BL21-OG2-intein2 was cultured in 1l medium and induced with 0.1 mM IPTG at 30°C for 2 h. The soluble OG2-intein2 protein accounted for 12.7% of the total soluble protein in the supernatant (Figure 4(a), lane SP). The final yield of OG2-intein2 was approximately 75 mg/L. About 85% of the OG2-intein2 was released and showed the band corresponding to the fusion partner on the gel (Figure 4(a), lane F). A single band corresponding to the molecular weight of the OG2 was also observed (Figure 4(a), lane P). The released OG2 was up to 95% after single-step chitin purification, and little band corresponding to the fusion partner was observed (Figure 4(a), lane P). The released OG2 was desalted using Sephadex G10 and lyophilized. Over 2 mg of OG2 was obtained from a 1l culture.

### 3.5. Antimicrobial Assay

The minimal inhibition concentration of expressed OG2 against *E. coli* K88 was 20 *μ*g/mL while that of the chemically synthesized OG2 was 16 *μ*g/mL. Both 100 *μ*g of expressed OG2 and 100 *μ*g of chemically synthesized OG2 showed similar halos in the agar diffusion test, indicating their similar antimicrobial activity (Figure 4(b)).

## 4. Discussion

AMPs are usually expressed as fusion proteins in *E. coli* to overcome their cytotoxicity in the production host. The expression of soluble fusion proteins is preferable to the expression of proteins that form inclusion bodies because the downstream processing is more convenient. Many reports have documented the expression of soluble AMPs with different fusion partners such as TrxA, SUMO, intein, ubiquitin, glutathione S-transferase, and maltose-binding protein [9–12]. The former four partners are preferred because of their small molecular weights, which result in a high ratio of small peptide to fusion protein. The optimization of growth conditions, such as the temperature and the inducer concentration, could also improve protein solubility and expression. In this study, OG2 was successfully expressed as a fusion protein with intein1 and intein2. A low induction temperature and a low IPTG concentration enhanced the solubility of OG2-intein2 and intein1-OG2.

Compared with the SUMO-, TrxA-, and ubiquitin-fusion systems, the intein system is more cost-effective because the target peptide can be isolated in a single chromatographic step and the process does not require the use of exogenous proteases. Although in vivo autocleavage is sometimes a drawback of the intein system [13], there was no observable in vivo autocleavage in this study. The amino acid closest to the cleavage site is one of the most important factors affecting the cleavage efficiency. The majority of the OG2-intein2 protein was released under the condition of 100 mM DTT, indicating that the C-terminal threonine in OG2 favored this cleavage. In contrast, the N-terminal lysine of OG2 inhibited the cleavage of intein1-OG2, consistent with our previous finding that low cleavage ratio of TrxA-EK-OG2 occurred after enterokinase digestion. Furthermore, the OG2 released from OG2-intein2 and the chemically synthesized OG2 exhibited similar antimicrobial activity against *E. coli* K88, indicating that the additional N-terminal methionine had little effect on the antimicrobial activity of OG2.

In conclusion, we developed a cost-effective method for the production of OG2, which is difficult to express in prokaryotic host strains and purification using conventional purification schemes. Further studies of the structure and antimicrobial mechanism of OG2 will be conducted in the near future.

## Figures and Tables

**Table 1:** Primers and restriction enzymes used for *OG2* amplification and vector construction.

Genes	Primers	Sequence 5′-3′	Restriction enzyme
*C-OG2* (C-terminal fusion)	PC1	GGGAATTCCATATGAAGAAATTCTTCCTGAAAGT GCT	*Nde *I
	PC2	CCCGCCACTTTGCAGCGAATTTTGGTCAGCACTT TCAGGAAGAAT	
	PC3	GGACTAGTGCATCTCCCGTGATGCAGGTACGAC AGCCACCCGCCACTTTGCAG	*Spe *I

*N-OG2* (N-terminal fusion)	PN1	CATAACTTTGTCGCGAATGACATCATTGTACACA ACAAGAAATTCTTCCTGAAAGTGCT	*Nru *I
	PN2	CCCGCCACTTTGCAGCGAATTTTGGTCAGCACTT TCAGGAAGAAT	
	PN3	CGCGGATCC **TTA**GGTACGACAGCCACCCGCCAC TTTGCAG	*Bam *HI

**Table 2:** Growth condition optimization of for the C-terminal fusion and N-terminal fusion strains.

Strains	Temperature (°C)	IPTG (mM)	Induction period (h)
BL21-OG2-intein2	37	0.5	2
	37	0.1	2
	30	0.1	2

BL21-intein1-OG2	37	0.5	2
	37	0.1	2
	30	0.1	2
	20	0.1	6
